# Remotely Controllable Liquid Marbles

**DOI:** 10.1002/adma.201201885

**Published:** 2012-07-26

**Authors:** Lianbin Zhang, Dongkyu Cha, Peng Wang

**Affiliations:** Water Desalination and Reuse Center, Chemical and Life Sciences and Engineering Division, King Abdullah University of Science and TechnologyThuwal 23955-6900, Saudi Arabia; Advanced Nanofabrication, Imaging and Characterization Core Lab, King Abdullah University of Science and TechnologyThuwal 23955-6900, Saudi Arabia

**Keywords:** liquid marbles, block copolymers, mesoporous materials, smart materials, surface chemistry, stimuli-responsive materials

With the ongoing need for miniaturized systems in many biological and chemical applications, there is an increasing demand for the development of versatile methods for controllable transport and manipulation of small volumes of liquids.^1–9^ Recently, liquid marbles — liquid droplets encapsulated by self-organized hydrophobic particles at the liquid/air interface — have been revealed as a new and effective approach for manipulating liquid droplets and compartmentalizing reactions in droplets.^1^, ^10–20^ Liquid marbles are considered as a perfect non-wetting system and they behave as micro-reservoirs of liquid capable of moving quickly without any leakage, because the hydrophobic particles on the liquid surface form nonstick droplet/substrate interfaces to reduce motion resistance.^10^, ^11^, ^17^ At the same time, the encapsulating particles on the liquid surface reduce evaporation of the encapsulated liquids.^11^, ^18^ These desirable characteristics make liquid marbles ideal platforms for storing and manipulating liquid droplets. Controllable movement of liquid marbles has been successfully achieved using various methods.^10^, ^11^, ^13^, ^14^ However, for practical applications, liquid marbles that allow both remote control of their movement and, more importantly, remotely triggerable opening are highly desirable,^19^, ^20^ but their fabrication is largely unexplored.

In this Communication we report remotely controllable liquid marbles that rupture upon ultraviolet (UV) illumination and can be remotely manipulated by an external magnetic field. The liquid marbles are prepared by encapsulating water droplets with novel core/shell-structured stimuli-responsive magnetic particles (designated RMPs), consisting of a silica-coated magnetite core and a mesoporous silica shell grafted with the responsive block copolymer (BCP) poly(2-vinylpyridine-*b*-dimethylsiloxane) (P2VP-*b*-PDMS). The silica shell is loaded with photoacid generator (PAG). When the RMP-based liquid marbles are exposed to UV illumination, acid (i.e., H^+^) is locally generated by the PAG, which in turn protonates the adjacent P2VP blocks on the RMP shell and thus induces a hydrophilic transition of the RMPs, leading to remotely triggered rupture of the liquid marbles. The current study provides an effective and convenient strategy for remotely manipulating droplets for many potential applications.

The preparation of the RMPs is schematically illustrated in **Figure** **1**a. First, uniform magnetite particles were prepared by means of a well-known solvothermal method^21^ and then they were coated with a thin and nonporous dense silica layer by a sol-gel approach to yield nonporous silica-coated Fe_3_O_4_ particles (i.e., Fe_3_O_4_@nSiO_2_).^22^ The dense silica layer on the surface of the Fe_3_O_4_ particles should protect the iron oxide core from leaching under acidic circumstances. Next, a mesoporous silica shell was coated on the Fe_3_O_4_@nSiO_2_ core by a surfactant-templating sol-gel approach to form the core/shell-structured Fe_3_O_4_@nSiO_2_@mSiO_2_ particles, using the surfactant hexadecyltrimethylammonium bromide (CTAB) as a template, followed by the removal of CTAB by a solvent-extracting process.^22^, ^23^ Finally, the mesoporous silica shell was functionalized with bromoalkyl groups by a silanization process using (3-bromopropyl)trimethoxysilane (BPS), and the responsive BCP P2VP-*b*-PDMS was then grafted onto the external surface of the mesoporous silica shell by means of a quaternization between the pyridyl groups on the BCP and the bromoalkyl groups on the silica shell of the Fe_3_O_4_@nSiO_2_@mSiO_2_,^24–26^ resulting in the RMPs. Because the BCP grafting is via the pyridyl groups on the P2VP blocks, the grafted BCP layer can be considered as a mixed polymer brush of P2VP and PDMS chains with broad distribution.^25^

**Figure 1:**
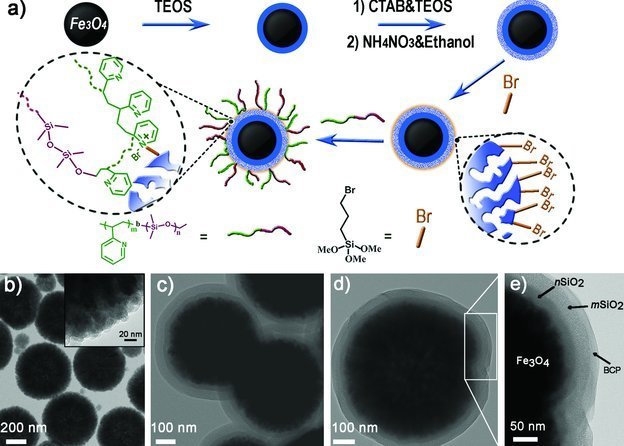
a) Schematic showing the preparation strategy for core/shell structured responsive magnetic particles. b–e) TEM images of Fe_3_O_4_ particles (b), Fe_3_O_4_@nSiO_2_@mSiO_2_ particles (c), and RMPs (d,e). Inset in (b): Enlarged TEM image of a single Fe_3_O_4_ particle. TEOS: Tetraethyl orthosilicate.

As shown in the transmission electron microscopy (TEM) image in Figure 1b, the as-prepared Fe_3_O_4_ particles had a mean diameter of ∼450 nm and were actually aggregates of nanoparticles with a diameter of ∼15 nm, giving rise to superparamagnetic behavior of the Fe_3_O_4_ particles.^21^, ^22^ Coating the Fe_3_O_4_ particles with the silica shells led to Fe_3_O_4_@nSiO_2_@mSiO_2_ particles with a dense nonporous silica middle layer and a mesoporous shell layer, the layer thicknesses being ∼20 and ∼40 nm, respectively (Figure 1c and Figure S1 in the Supporting Information). After silanization with BPS and BCP grafting, a rim-like coating with a thickness of ∼15 nm was formed on the surface of the Fe_3_O_4_@nSiO_2_@mSiO_2_ particles (Figures 1d,e), indicating the successful grafting of the P2VP-*b*-PDMS on the exterior of the mesoporous silica shell. At the same time, the successful grafting of the BCP was also confirmed by Fourier transform infrared (FTIR) spectra measurements (Figure S2 in the Supporting Information). The amount of grafted BCP on Fe_3_O_4_@nSiO_2_@mSiO_2_ particles was estimated by thermogravimetric analysis (TGA) measurements to be 1.9% (Figure S3 in the Supporting Information). N_2_ sorption–desorption isotherms of Fe_3_O_4_@nSiO_2_@mSiO_2_ and BCP-grafted Fe_3_O_4_@nSiO_2_@mSiO_2_ exhibited typical type-IV curves (Figure S4 in the Supporting Information). The Brunauer–Emmett–Teller (BET) surface area and average pore size of Fe_3_O_4_@nSiO_2_@mSiO_2_ were 436 m^2^ g^−1^ and 2.1 nm, respectively. For the BCP-grafted Fe_3_O_4_@nSiO_2_@mSiO_2_, these parameters were 330 m^2^ g^−1^ and 2.0 nm, respectively. These results indicate that the grafted BCP was predominantly present on the exterior of the silica shell and the mesopores of the silica shell remained accessible after the silanization and BCP grafting, which could be used for incorporation of functional molecules.^27^

The BCP P2VP-*b*-PDMS comprises blocks of pH-responsive P2VP and hydrophobic PDMS. As has been reported, owing to its pH-responsive property, P2VP can alter its wettability as well as its conformation by protonation and deprotonation of its pyridyl groups in response to pH changes,^26^, ^28^, ^29^ and thus the grafted BCP endows the core/shell-structured particles with a pH-responsive property. Dynamic light scattering (DLS) and zeta potential measurements were first employed to characterize the pH-responsive property of the as-prepared RMPs in water with different pH values. As shown in **Figure** **2**a, the RMPs could be well dispersed in water at ≤pH 2.0 and in these acidic aqueous media, the RMPs had an average zeta potential of ca. +20 mV and a hydrodynamic diameter of ca. 600 nm, which is comparable to that of the single particle in the TEM images (Figure 1). As the pH of the aqueous media increased above 3.0, the RMPs aggregated seriously and they predominantly stayed at the water/air interface (Figure 2b). It should be noted that when the pH of the water was greater than 4.0, the RMPs aggregated so seriously that they predominantly stayed at the water/air interface and thus became unstable in the water, which made the sample not suitable for the DLS and zeta potential measurements.

**Figure 2:**
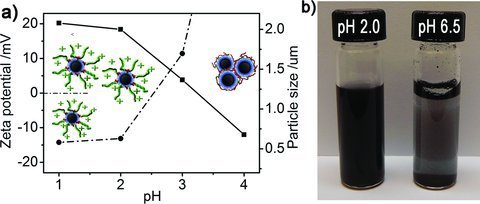
a) Hydrodynamic diameter (dotted-dashed line) and zeta potential (solid line) of the RMPs as a function of the pH of the water. b) Digital photograph showing the dispersion of the RMPs in water of pH 2.0 and 6.5.

As a weak polybase, P2VP has a p*K*_a_ of about 3.6^28^ and thus when dispersed in the acidic water (≤pH 2.0), the P2VP chains on the surface of Fe_3_O_4_@nSiO_2_@mSiO_2_ particles become protonated and charged, which increases the amount of positive charges on the particles as demonstrated by the zeta potential plot. The repulsion between the positively charged particles results in the stable dispersion of the RMPs in the acidic water (≤pH 2.0). When the pH of the aqueous media is higher than 3.0, owing to the deprotonation of most of the pyridyl groups on the P2VP blocks the electrostatic repulsion decreases while the attractive hydrophobic force between the particles dominates, leading to inevitable particle aggregation at high pH. Because of their high hydrophobicity, the aggregated RMPs stayed at the water/air interface at high pH. Moreover, the aggregation–redispersion of the RMPs was highly reversible and could be tuned by alternating the pH between 6.5 and 2.0 for many cycles.

Contact angle (CA) measurements were also conducted to probe the responsive wettability of the RMPs (Figure S5 in the Supporting Information). The PDMS block of the BCP on the RMPs has a desirably low glass transition temperature (*T*_g_) of about –62 °C (according to the supplier), and thus it can be considered as a liquid-like polymer with high flexibility at ambient temperature. In air, the liquid-like PDMS, which is more hydrophobic than P2VP, can spontaneously move to the exterior of the grafted copolymer layer and dominantly expose itself to air, making the surface of RMP hydrophobic.^26^ It was observed that a water droplet of pH 6.5 formed a sphere with a CA of 157.2° on a thin layer of RMPs, which has a rough surface because of the stacking of the RMPs. The rough surface structures amplified the surface wetting behavior, resulting in superhydrophobicity. Although the contact angle measurements on the rough RMP-formed thin layer do not directly reflect the surface wetting behavior of individual RMPs, they can still be used to indicate the tendency of the hydrophilic transition of the RMPs when in contact with an acidic water droplet (detailed discussion can be found in the Supporting Information). When a water droplet of pH 2.0 was used, a CA of 135.2° was obtained under otherwise the same conditions, indicating that the RMP-formed thin layer exhibited relatively hydrophilic behavior owing to the protonation of the P2VP blocks with a water droplet of pH 2.0. Thus, the responsive behavior of the P2VP blocks on the BCP-grafted RMPs allows precise regulation of the dispersibility and wettability of these particles, which holds promise for controllable manipulation of liquid marbles based on BCP-grafted RMPs.

Because of the high hydrophobicity of the RMPs, it was possible to prepare liquid marbles simply by rolling water droplets (pH 6.5) on a pile of the RMPs. As the water droplets rolled, the RMPs spontaneously self-organized at the water/air interface, encapsulating the water droplets and rendering the droplets non-wetting to the substrate (**Figure** **3**a). The as-prepared liquid marbles were stable and could be readily handled by tweezers without breaking up. Figures 3b and c show that the liquid marbles remained intact after being transferred onto a glass substrate and the surface of water (pH 6.5) in a Petri dish.

**Figure 3:**
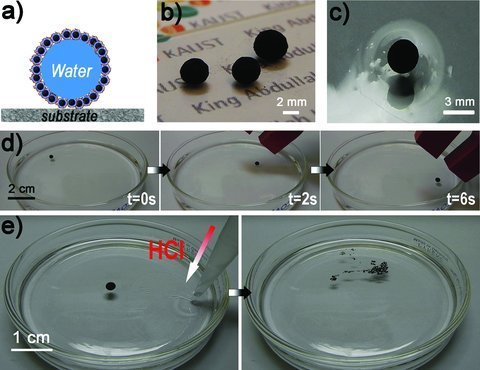
a) Schematic showing a liquid marble composed of an encapsulated water droplet and hydrophobic RMPs. b,c) Digital photographs showing the liquid marbles placed on a glass slide (b) and water surface (c). The liquid marbles were prepared using 10–15 μL water of pH 6.5. d) Snapshots showing controlled movement of the liquid marble floating on the water surface in response to an external magnetic field. e) Snapshots showing the rupture of the liquid marble (15 μL) placed on the water surface after addition of concentrated HCl. The final pH of the solution in the Petri dish is ∼1.8.

The RMPs showed superparamagnetic behavior with a high saturation magnetization of 26.1 emu g^−1^ (Figure S6 in the Supporting Information). As a result, the RMPs enable the liquid marbles to be remotely manipulated by an external magnetic field. As illustrated in Figure 3d (Video S1 in the Supporting Information), a liquid marble was first placed on the water surface (pH 6.5), and then a macroscopic magnet was used as an external manipulator to remotely move the liquid marble around on the water surface. The liquid marble not only moved quickly (with a speed of ∼3 cm s^−1^) under the external manipulator, but also was able to change its direction of movement in response to the motion of the magnet.

The as-prepared liquid marbles with the encapsulated water droplet (pH 6.5) remained stable for several hours on the surface of bulk water with pH 6.5. The presence of the RMPs at the water/air interface of the liquid marbles prevents the diffusion of water between the interior of the marbles and the bulk water. However, when concentrated HCl was slowly added to the bulk water, the liquid marble ruptured immediately, with the final pH of the aqueous media being ∼1.8 (Figure 3e and Video S2 in the Supporting Information). As discussed above, when the pH of the bulk water decreases below a critical value, the P2VP chains on the RMPs that are in contact with the bulk water are protonated, which increases the hydrophilicity of the RMPs and leads to the partial dispersion of the RMPs, resulting in a violent rupture of the marbles. Furthermore, a series of bulk water samples with various pH values was used to test the stability of the liquid marbles placed on the surface of these water samples and the lifetime of the liquid marbles (with pH 6.5 for the encapsulated water droplets) was recorded. For acidic bulk water (≤ pH 2.0), the lifetime of the liquid marbles was less than 10 s, and the liquid marbles quickly ruptured upon contact with the water. With an increase of the pH of the bulk water, the lifetime of the liquid marbles increased dramatically. For water with pH 3.0, the liquid marble remained stable for about 1.5 h. As the pH of the bulk water increased above 4.0 (specifically 4.0, 6.5, and 8.0), the liquid marbles remained stable for more than 12 h (at a relative humidity of ∼90%) without breakage or significant size shrinkage, which can be attributed to the reduced evaporation of the encapsulated water. These results demonstrate the pH-responsive property of the RMP-based liquid marbles, which could be potentially useful for on-demand release of materials encapsulated inside the liquid marbles.

Although we can readily disintegrate the liquid marbles by changing the pH of the bulk water, which also has been reported by Fujii and co-workers,^19^, ^20^ for many applications the addition of concentrated acid to lower the system pH is still inconvenient or even problematic. Therefore, a more effective and controllable strategy to trigger the rupture of the RMP-based liquid marbles is practically desirable. In the current system, a PAG, (4-phenoxyphenyl)diphenylsulfonium triflate, was loaded into the accessible mesopores in the shells of the RMPs, and remotely UV-triggered rupture of the liquid marbles prepared from the PAG-loaded RMPs was realized. The PAG has an absorption peak at ca. 270 nm and illumination around this wavelength generates strong acid (i.e., H^+^).^30^ The PAG was first loaded into the mesoporous silica shell of the RMPs by soaking the RMPs in a dichloromethane solution of PAG. The PAG loading was predominantly by physical adsorption. Since dichloromethane is a good solvent for both the P2VP and PDMS blocks, these chains should adopt an extended conformation during PAG loading and which would enable the entry of the PAG into the mesopores. The PAG-loaded RMPs were then magnetically separated and washed with dichloromethane before drying. Upon drying, the polymer chains will collapse and the collapsed polymer chains will seal the PAG within the mesoporous silica shell. The PAG-loaded RMPs were subsequently used for the preparation of liquid marbles and one thus-prepared liquid marble was then transferred to the surface of water (pH 6.5) in a Petri dish. As a comparison, a liquid marble prepared from the RMPs without PAG loading was also placed on the same water surface. As shown in **Figure** **4**a (also in Video S3 in the Supporting Information), after ∼20 s of UV illumination, the liquid marble prepared from the PAG-loaded RMPs ruptured, while the one without PAG loading remained stable even after prolonged UV illumination. After the rupture of the liquid marble, the pH of the bulk water (∼15 mL) did not show a significant decrease. As illustrated in Figure 4b, upon UV illumination, the PAG within the mesoporous silica shell of the RMPs generates a locally high concentration of H^+^, which induces protonation of the adjacent P2VP chains on the particle surface and causes a hydrophilic transition of the RMPs, leading to the rupture of the liquid marble. Thus, the loading of PAG into the mesoporous shells of the RMPs provides an effective way of rupturing the liquid marbles by remote light-triggering by locally changing the pH of individual marbles, and thus avoids the trouble of changing the pH of the bulk water, which is very favorable for the many practical applications.

**Figure 4:**
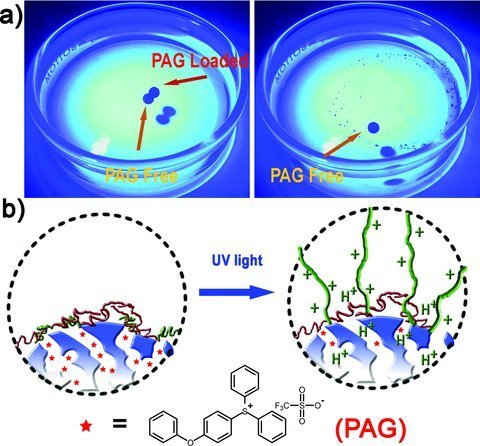
a) Snapshots showing the UV-triggered rupture of the liquid marble prepared from PAG-loaded RMPs. As a comparison, the liquid marble prepared from PAG-free RMPs remained unchanged even after extended UV illumination. b) Schematic showing the UV-triggered hydrophilic transition of the PAG-loaded RMPs.

In conclusion, we have demonstrated remotely controllable liquid marbles prepared from novel core/shell-structured responsive magnetic particles. The loading of PAG into the mesopores of RMPs enables the thus-resulting liquid marbles to rupture upon UV illumination and to be readily controlled by an external magnetic field. We envision that the potential applications of such remotely controllable liquid marbles will include smart delivery of water-soluble agents to initiate chemical reactions on demand, channel-free microfluidic systems,^3^ and sensors with visual indication capability.^11^

## Experimental Section

*Preparation of the RMPs:* The Fe_3_O_4_, Fe_3_O_4_@nSiO_2_, and Fe_3_O_4_@nSiO_2_@mSiO_2_ particles were prepared according to well-established literature methods (details can be found in the Supporting Information). The block copolymer P2VP-*b*-PDMS was grafted onto the exterior of Fe_3_O_4_@nSiO_2_@mSiO_2_. Briefly, the Fe_3_O_4_@nSiO_2_@mSiO_2_ particles were soaked in a 1% anhydrous toluene solution (10 mL) of BPS for 20 min at 70 °C to functionalize the surface of the particles with bromoalkyl groups by silanization. The silanized particles were washed with toluene and ethanol to remove the unreacted silanes. The BPS-functionalized particles were then dispersed in 10 mL of a 1% solution of the block copolymer (P2VP-*b*-PDMS) in nitromethane (NM) and dimethyl sulfoxide (DMSO) (1:1, v:v). The mixture was stirred at 60 °C for 60 h. The particles with the grafted BCP layer were separated by a magnet and washed repeatedly with dichloromethane. Finally, the BCP-grafted Fe_3_O_4_@nSiO_2_@mSiO_2_ particles (RMPs) were dried at 60 °C under vacuum for 12 h.

*Loading of PAG into the mesoporous shell of the RMPs:* Loading of PAG into the RMPs was realized by soaking 0.1 g of the particles in a dichloromethane solution (20 mL) of PAG (20 mM L^−1^) for 6 h. The PAG-loaded RMPs were then magnetically separated, washed with dichloromethane, and dried in vacuum at 60 °C for 2 h.

*Preparation of liquid marbles:* Water droplets (10–15 μL) were carefully deposited onto a pile of the RMP (or PAG-loaded RMP) powder with a micropipet. Gentle rolling of the droplet on the powder bed led to entire encapsulation of the liquid by the RMPs, resulting in a liquid marble. The thus-resulting liquid marbles were then transferred readily with tweezers onto a glass substrate or the surface of water (pH 6.5) in a Petri dish for further analysis.
