# Supplementary material for: Remotely Controllable Liquid Marbles
Source: Adv Mater. 2012 Jul 26;24(35):4756–60. doi: 10.1002/adma.201201885 (PMC3546392; doi:10.1002/adma.201201885)
Supplement: Supplementary file 4 [file adma0024-4756-SD4.pdf]

Copyright WILEY-VCH Verlag GmbH & Co. KGaA, 69469 Weinheim, Germany, 2012.

ADVANCED MATERIALS

# Supporting Information

for *Adv. Mater.*, DOI: 10.1002/adma.201201885

## Remotely Controllable Liquid Marbles

*Lianbin Zhang, Dongkyu Cha, and Peng Wang**

Supporting Information For

# Remotely Controllable Liquid Marbles

By *Lianbin Zhang, Dongkyu Cha, and Peng Wang**

Chemical and Life Sciences & Engineering Division, Water Desalination and Reuse Center
King Abdullah University of Science and Technology,
Thuwal 23955–6900 (Saudi Arabia)
E-mail: peng.wang@kaust.edu.sa

**This file includes Experimental details, Supplementary Figure S1-S6, Legends for Supplementary Information Multimedia Video S1-S3, and Supplementary References.**

**Experimental details:**

***Materials.*** Poly(2-vinylpyridine-*b*-dimethylsiloxane) block copolymer P5321 (P2VP-*b*-PDMS 16,000-*b*-10,000 g/mol) was purchased from Polymer Source, Inc., Canada. Tetraethyl orthosilicate (TEOS), (3-bromopropyl)trimethoxysilane (BPS), cetyltrimethylammonium bromide (CTAB), $FeCl_3·6H_2O$, (4-phenoxyphenyl)diphenylsulfonium triflate (PAG), ammonia aqueous solution (28 wt.%), $NH_4NO_3$, ethylene glycol, anhydrous toluene, ethanol, nitromethane (NM), and dimethyl sulfoxide (DMSO) were purchased from Sigma-Aldrich. All these chemicals were used as received. Deioned (DI) water purified in a Milli-Q (Millipore) system was used in all experiments.

***Synthesis of $Fe_3O_4$ particles.*** The magnetite particles were prepared through a solvothermal reaction according to a previous report.[1] Briefly, 2.70 g of $FeCl_3·6H_2O$ and 7.20 g of sodium acetate were dissolved in 100 mL of ethylene glycol. The obtained homogeneous solution was solvothermally heated at 200 °C for 8 h. The obtained black particles were washed with ethanol and water for 6 times, and then dried at 60 °C under vacuum for 12 h.

***Synthesis of $Fe_3O_4$@n$SiO_2$@m$SiO_2$ particles.*** The core-shell $Fe_3O_4$@n$SiO_2$@m$SiO_2$ particles were prepared according to the previously reported method.[2] Briefly, 0.10 g of the previously prepared $Fe_3O_4$ particles (~450 nm in diameter) were treated with 0.1 M HCl aqueous solution (50 mL) by ultrasonication for 5 min. The magnetite particles were magnetically separated, washed with DI water, and then homogeneously dispersed in the mixture of ethanol (160 mL), DI water (40 mL) and concentrated ammonia aqueous solution (2.0 mL, 28 wt.%), followed by the addition of TEOS (0.5 mL) to obtain $Fe_3O_4$@n$SiO_2$. After stirring at room temperature for 6 h, the $Fe_3O_4$@n$SiO_2$ particles were separated, washed with ethanol and water, and then re-dispersed in a mixed solution containing CTAB (0.3 g), water (80 mL), concentrated ammonia aqueous solution (1.0 mL, 28 wt.%), and ethanol (60 mL). The mixed solution was homogenized for 0.5 h to form a uniform dispersion, and then 0.5 mL of TEOS was added dropwise to the dispersion under a continuous stirring. The mixture was left under stirring for 12 h before the product was collected with a magnet and washed repeatedly with ethanol and water to remove nonmagnetic by-products. Finally, the purified particles were dispersed in 60 mL of $NH_4NO_3$ in ethanol solution (6.0 g/L) and refluxed at 60 °C for 1 h to extract the CTAB surfactant.[3] This extraction process was repeated three times. After centrifugation, washing with ethanol, and then drying at 60 °C under vacuum for 12 h, the $Fe_3O_4$@n$SiO_2$@m$SiO_2$ particles were finally produced.

***Block copolymer (BCP) grafting on the $Fe_3O_4$@$nSiO_2$@$mSiO_2$ particles to prepare the RMPs.*** Weighed amount of 0.1 g of the $Fe_3O_4$@$nSiO_2$@$mSiO_2$ particles were immersed in a 1% anhydrous toluene solution (10 mL) of BPS for 20 minutes at 70 °C to functionalize the surface of the particles with bromoalkyl groups via silanization. A short reaction time is favorable for the attachment of the functional group to the external surfaces of the mesoporous silica shell rather than the interior of the mesopores.[4] The silanized particles were washed with toluene and ethanol to remove the unreacted silanes. The BPS-functionalized particles were then dispersed in 10 mL of 1% solution of block copolymer (P2VP-*b*-PDMS) in nitromethane (NM) and dimethyl sulfoxide (DMSO) (1:1, v:v). The mixture was stirred at 60 °C for 60 h. The particles with the grafted polymer layer were separated by a magnet and washed repeatedly with dichloromethane. Finally, the BCP-grafted $Fe_3O_4$@$nSiO_2$@$mSiO_2$ particles (RMPs) were dried at 60 °C under vacuum for 12 h.

***Characterization.*** Transmission electron microscopy (TEM) observations were carried out on FEI Titan ST instrument operated at 300.0 kV. $N_2$ sorption at 77 K was carried out with a Micromeritics Tristar II 3020 surface and porosimetry analyzer, and the surface area was evaluated using the BET method. Before measurements, the samples were degassed under vacuum at 100 °C for at least 6 h. By using the Barrett-Joyner-Halenda (BJH) model, the mesopore size distributions were derived from the adsorption branches of isotherms. Fourier-transform infrared (FTIR) spectra were collected on Nicolet Fourier spectrophotometer using KBr pellets. The amount of the grafted BCP on the RMPs was characterized by thermalgravimetric analysis (TGA) measurements, which were carried out on a TG 209 F1 (NETZSCH) instrument with a heating rate of 10 °C/min. The magnetization curves were obtained by vibrating-sample magnetometer (VSM, LakeShore7037/9509-P). The hydrodynamic diameter and zeta potential studies of the RMPs in water with different pH were carried out on a Malvern Nano-ZS zetasizer at room temperature. The CA measurements were performed with an Attension Theta system (KSV Instruments Ltd., Finland) at room temperature. Water droplets of ~4.0 μL with pH of 6.5 and 2.0 were dropped carefully onto a thin bed of the RMPs. An average contact angle value was obtained by measuring the same sample at three different positions. UV illumination was produced by a Dymax BlueWave 200 UV Curing Spot Lamp, connected with a UV light guide positioned ~5 cm above the liquid marbles. Digital images and videos were captured by using a Nikon camera (Nikon COOLPIX P100).

**Supplementary Figures:**

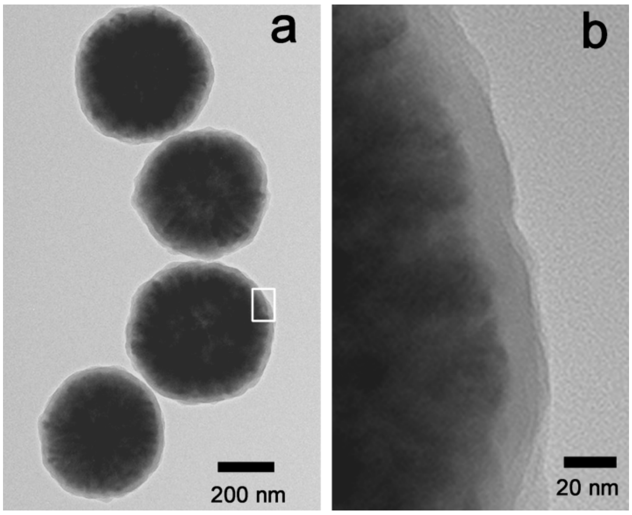


***Figure S1.*** TEM images of the $Fe_3O_4$@n$SiO_2$ particles.

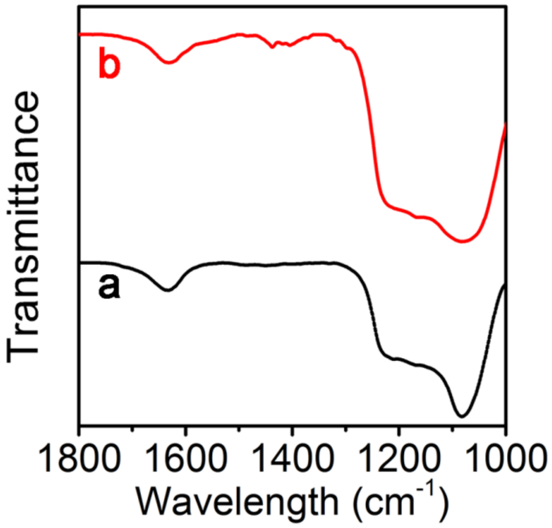


***Figure S2.*** FTIR spectra of $Fe_3O_4$@n$SiO_2$@m$SiO_2$ (a) and RMPs (b). The successful grafting of BCP onto $Fe_3O_4$@n$SiO_2$@m$SiO_2$ was confirmed by the appearance of the band at 1434 $cm^{-1}$ in the FTIR spectra, which is characteristic of the pyridine group of P2VP.[5]

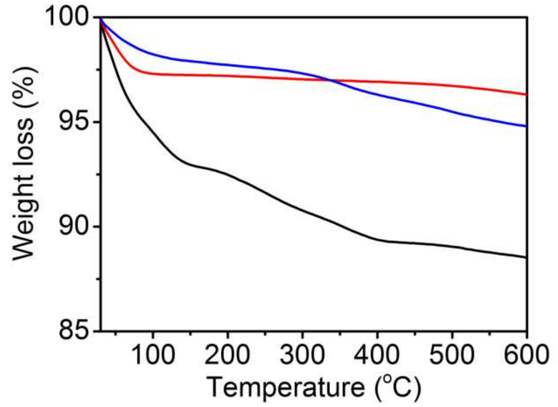


***Figure S3.*** TGA curves of $Fe_3O_4$@n$SiO_2$@m$SiO_2$ (red), silanized $Fe_3O_4$@n$SiO_2$@m$SiO_2$ with BPS (blue), and RMPs (black).

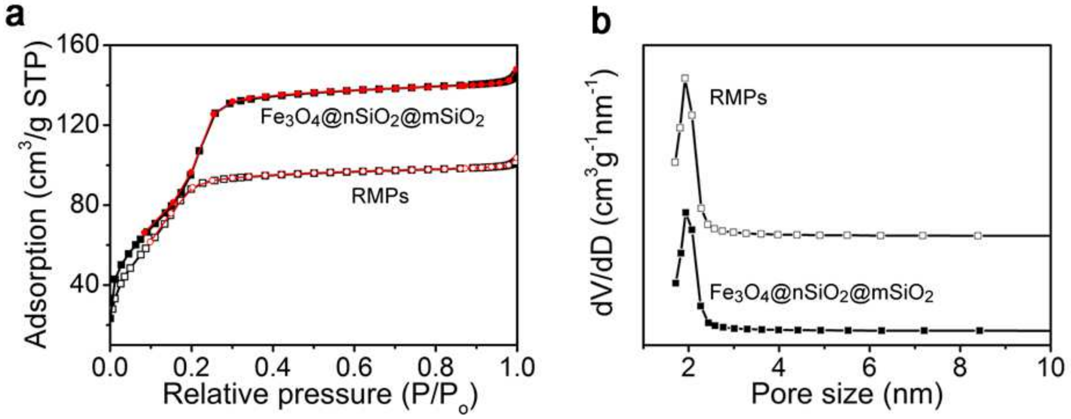


***Figure S4.*** $N_2$ adsorption–desorption isotherms (a) and mesopore size distribution (b) of $Fe_3O_4$@n$SiO_2$@m$SiO_2$ and RMPs.

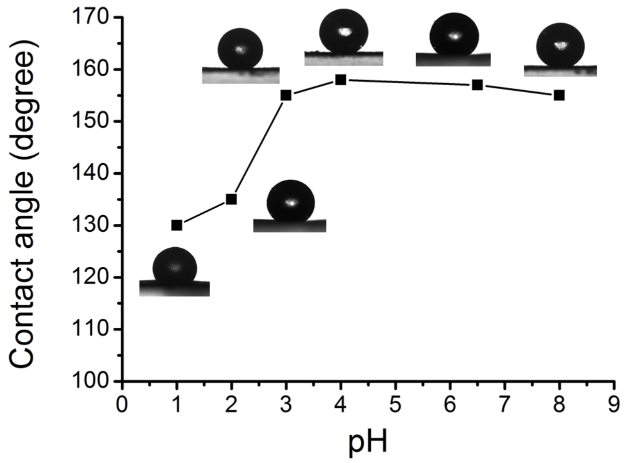


***Figure S5.*** Contact angle of water droplets on the RMPs-formed thin bed as a function of the droplet pH. Insets: The shapes of the water droplets (~4.0 μl) with different pH on the surface of the thin bed of RMPs. Our results showed that the water contact angles for the droplets

with a pH ≥ 3.0 were greater than 150°, indicating the superhydrophobic property for the surface. As is well-known, surface roughness has a significant influence on the surface wetting behavior, and therefore the roughness of the thin bed of RMPs further amplified the wetting behavior of the hydrophobic RMPs, resulting in the superhydrophobicity.

However, for the droplets with a pH ≤ 2.0, the contact angles decreased, indicating the relatively hydrophilic transtion of the surface of the RMPs-formed thin bed. As the pH of the water droplets decreases below a certain level (in this case pH < 3.0), the P2VP chains on the RMPs that are in contact with droplets are protonated and exhibit an extended conformation because of the electrostatic repulsion among the like charges. They then stretch to the exterior of the grafted block copolymer layer, and with the protonated P2VP exposed on the surface, the surface of the RMPs acquire hydrophilic property, as evidenced by the DLS and zeta potential measurements (Figure 2). It is this hydrophilic transition of the individual RMPs in response to the decrease of pH that results in the decrease in the water contact angle on the RMP-formed thin bed (e.g., 130° for pH 1.0 and 135° for pH 2.0). It should be noted that the surface of the RMPs-formed thin bed is still hydrophobic (contact angle > 90°), although the contact angle decreased for the acidic droplets (pH ≤ 2.0).

These seemingly conflicting observations can be explained by the following: First, the surface wetting behavior of the RMPs thin bed with a rough surface is in the Cassie state, where the acidic droplets may be suspended on the top of the rough particle-stacked surface with interspersed air pockets. In this case, although individual RMPs that in contact with the acidic droplets acquire hydrophilic property, the air trapped beneath the water imparts the rough surface actually hydrophobic. Secondly, because the thin bed is formed by the loose stacking of the RMPs, the RMPs can easily absorb onto the surface of the applied water droplets upon contact, which would then restrict the diffusion and spread of water over the thin bed surface, leading to a relative high water contact angle on the RMPs-formed thin bed even for water droplet with low pH (e.g., 1.0 and 2.0). Based on these considerations, the contact angles on the rough RMPs-formed thin bed do not directly reflect the surface wetting behavior of individual RMPs. However, the contact angle measurements can still be used to indicate the tendency of the hydrophilic transition of the RMPs when contact with acidic water droplet.

In summary, although the water contact angle on the RMPs-formed thin bed was measured to be 135° and 130° for water droplets with a pH of 2.0 and 1.0, individual RMPs became hydrophilic. It is this hydrophilic transition of the individual RMPs in response to the decrease of pH that lead to the rupture of the liquid marbles.

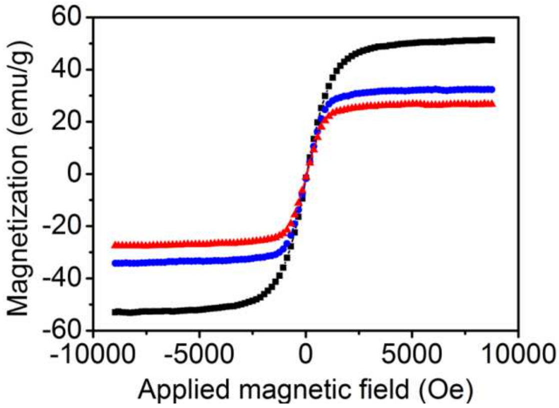


***Figure S6.*** Vibrating sample magnetization (VSM) curves of $Fe_3O_4$ (black), $Fe_3O_4$@n$SiO_2$@m$SiO_2$ (blue), and RMPs (red). The saturation magnetizations of $Fe_3O_4$, $Fe_3O_4$@n$SiO_2$@m$SiO_2$, and RMPs are 52.5, 32.3, and 26.1 emu/g, respectively. All the curves showed no hysteresis, remanence, and coercivity, indicating the superparamagnetism of these particles.

**Legends for Supplementary Information Multimedia Video S1-S3:**

***Video S1.*** Controlled movement of a liquid marble placed on water surface under the magnetic field.

***Video S2.*** Rupture of a liquid marble placed on the water surface after the addition of the concentrated HCl.

***Video S3.*** UV-triggered rupture of the liquid marble prepared from the PAG-loaded RMPs. As a comparison, a liquid marble prepared from the PAG-free RMPs was also placed on the water surface.

**Supplementary Reference:**

[1] P. Wang, Q. H. Shi, Y. F. Shi, K. K. Clark, G. D. Stucky, A. A. Keller, *J. Am. Chem. Soc.* **2009**, *131*, 182.

[2] Y. H. Deng, D. W. Qi, C. H. Deng, X. M. Zhang, D. Y. Zhao, *J. Am. Chem. Soc.* **2008**, *130*, 28.

[3] J. P. Yang, F. Zhang, Y. R. Chen, S. Qian, P. Hu, W. Li, Y. H. Deng, Y. Fang, L. Han, M. Luqman, D. Y. Zhao, *Chem. Commun.* **2011**, *47*, 11618.

[4] R. Liu, P. H. Liao, J. K. Liu, P. Y. Feng, *Langmuir* **2011**, *27*, 3095.

[5] J. Q. Lu, S. S. Yi, *Langmuir* **2006**, *22*, 3951.
